# A Novel RP-UHPLC-MS/MS Approach for the Determination of Tryptophan Metabolites Derivatized with 2-Bromo-4′-Nitroacetophenone

**DOI:** 10.3390/biomedicines12051003

**Published:** 2024-05-02

**Authors:** Timotej Jankech, Ivana Gerhardtova, Petra Majerova, Juraj Piestansky, Lubica Fialova, Josef Jampilek, Andrej Kovac

**Affiliations:** 1 Institute of Neuroimmunology, Slovak Academy of Sciences, Dubravska Cesta 9, 845 10 Bratislava, Slovakia; timotej.jankech@gmail.com (T.J.); ivka.gerhardtova@gmail.com (I.G.); petra.majerova@savba.sk (P.M.); piestansky@fpharm.uniba.sk (J.P.); lubica.fialova@savba.sk (L.F.); 2 Department of Analytical Chemistry, Faculty of Natural Sciences, Comenius University Bratislava, Ilkovicova 6, 842 15 Bratislava, Slovakia; 3 Department of Galenic Pharmacy, Faculty of Pharmacy, Comenius University Bratislava, Odbojarov 10, 832 32 Bratislava, Slovakia; 4 Department of Pharmacology and Toxicology, University of Veterinary Medicine and Pharmacy in Kosice, Komenského 68/73, 041 81 Kosice, Slovakia

**Keywords:** derivatization, liquid chromatography, Alzheimer’s disease, tryptophan metabolites

## Abstract

Many biologically active metabolites of the essential amino acid L-tryptophan (Trp) are associated with different neurodegenerative diseases and neurological disorders. Precise and reliable methods for their determination are needed. Variability in their physicochemical properties makes the analytical process challenging. In this case, chemical modification of analyte derivatization could come into play. Here, we introduce a novel fast reversed-phase ultra-high-performance liquid chromatography (RP-UHPLC) coupled with tandem mass spectrometry (MS/MS) method for the determination of Trp and its ten metabolites in human plasma samples after derivatization with 2-bromo-4′-nitroacetophenone (BNAP). The derivatization procedure was optimized in terms of incubation time, temperature, concentration, and volume of the derivatization reagent. Method development comprises a choice of a suitable stationary phase, mobile phase composition, and gradient elution optimization. The developed method was validated according to the ICH guidelines. Results of all validation parameters were within the acceptance criteria of the guideline, i.e., intra- and inter-day precision (expressed as relative standard deviation; RSD) were in the range of 0.5–8.2% and 2.3–7.4%, accuracy was in the range of 93.3–109.7% and 94.7–110.1%, limits of detection (LODs) were in the range of 0.15–9.43 ng/mL, coefficients of determination (R2) were higher than 0.9906, and carryovers were, in all cases, less than 8.8%. The practicability of the method was evaluated using the blue applicability grade index (BAGI) with a score of 65. Finally, the developed method was used for the analysis of Alzheimer’s disease and healthy control plasma to prove its applicability. Statistical analysis revealed significant changes in picolinic acid (PA), anthranilic acid (AA), 5 hydroxyindole-3-acetic acid (5-OH IAA), and quinolinic acid (QA) concentration levels. This could serve as the basis for future studies that will be conducted with a large cohort of patients.

## 1. Introduction

**Figure 1:**
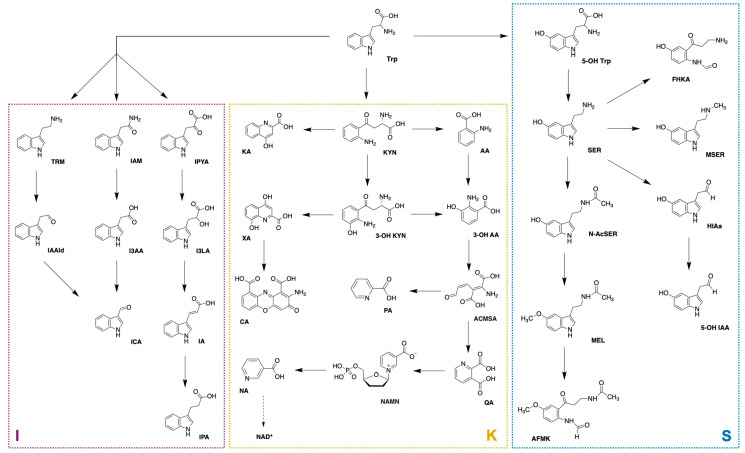
Tryptophan metabolism (I: indole pathway; K: kynurenine pathway; S: serotonin pathway): highlighted analytes were determined/investigated in the experimental part. Abbreviations: 3-OH AA: 3-hydroxyanthranilic acid; 3-OH KYN: 3-hydroxykynurenine; 5-OH IAA: 5 hydroxyindole-3-acetic acid; 5-OH Trp: 5-hydroxytryptophan; AA: anthranilic acid; ACMSA: 2-amino-3 carboxymuconic semialdehyde; AFMK: N-acetyl-N-formyl-5-methoxykynurenamine; CA: cinnabarinic acid; FHKA: formyl-5-hydroxykynurenamine; HIAa: 5-hydroxyindole-3-acetaldehyde; I3AA: indole-3-acetic acid; I3LA: indole-3-lactic acid; IA: indoleacrylic acid; IAAld: indole-3-acetaldehyde; IAM: indole-3-acetamide; ICA: indole-3-carboxaldehyde; IPA: indole-3-propionic acid; IPYA: indole-3-pyruvate; KA: kynurenic acid; KYN: kynurenine; MEL: melatonin; MSER: N-methylserotonin; N-AcSER: N acetylserotonin; NA: nicotinic acid; NAD+: nicotinamide adenine dinucleotide; NAMN: nicotinamide mononucleotide; PA: picolinic acid; QA: quinolinic acid; SER: serotonin; TRM: tryptamine; Trp: tryptophan; XA: xanthurenic acid.

L-tryptophan (Trp) is an essential amino acid precursor of many significant biologically active molecules [1,2]. Around 90–95% of absorbed Trp enters the kynurenine pathway (KP) [3,4], and a small amount (approximately 5% of Trp) enters the serotonin pathway (SP) [4,5]. The last way of Trp degradation is mainly performed by intestinal microorganisms producing indole-based metabolites in the indole pathway (IP) (Figure 1) [3]. Several metabolites formed in these pathways are neuroactive. For example, indole-3-propionic acid (IPA), indole-3-lactic acid (I3LA), and indole-3-acetic acid (I3AA) have neuroprotective properties, and additionally, they are involved in the modulation and regulation of various processes (i.e., inflammatory response modulation, CNS inflammation regulation) [6,7]. Kynurenic acid (KA) and picolinic acid (PA) are neuroprotective metabolites, while quinolinic acid (QA), 3-hydroxyanthranilic acid (3-OH AA), and 3-hydroxykynurenine (3-OH KYN) are among the neurotoxic ones [2,8,9]. Changes in the concentration of Trp metabolites are often associated not only with neurological disorders [10] and neurodegenerative diseases [11,12,13] (for example, Lewy body disorder [14], Huntington’s disease [15], and Alzheimer’s disease (AD) [16]) but also cardiovascular diseases (CVDs) [17] or cancer [12].

Many Trp metabolites play an important role in different diseases or disorders; thus, they can serve as biomarkers in pathogenesis. Furthermore, alterations in one pathway can have an influence on another pathway. Thus, it is crucial and challenging to develop reliable analytical methods for the simultaneous determination of Trp metabolites and for revealing the relationships between individual pathways [3,12,18]. In addition to capillary electrophoresis (CE) [19] or gas chromatography (GC) [20], liquid chromatography (LC) [15] is a prevalent analytical platform for the simultaneous determination of Trp metabolites in biological fluids (i.e., serum, plasma, cerebrospinal fluid (CSF), urine) or tissues [21,22,23]. Nowadays, high-performance liquid chromatography (HPLC) and ultra-high-performance liquid chromatography (UHPLC) in conjunction with ultraviolet (UV) [24], fluorescence (FLD) [25], electrochemical (ECD) [26] detection or, more preferably, mass spectrometry (MS) [15,22], can be used. The sensitivity and selectivity of the method can be improved using UHPLC with tandem MS (MS/MS) [27,28]. In addition to reversed-phase (RP)-LC mode, hydrophilic interaction chromatography (HILIC) can be applied in the analysis of Trp metabolites [29]. However, some analytical limitations remain, such as different concentration levels of these analytes, variability in their physicochemical properties, poor retention in the RP chromatographic system, or low ionization efficiency in the ESI ion source [30,31,32]. To overcome these limitations, chemical modification–derivatization can be applied prior to LC analysis [33,34]. Several derivatization-based methodologies using different types of reactions for Trp metabolite labeling have been recently proposed [35,36,37]. The biggest drawback of derivatization is that Trp metabolites do not have a common functional group, making the derivatization process more complicated [8]. One way to overcome this drawback is to split the sample into two equal parts and subject one of them to derivatization [35]. The second possibility is the use of derivatization reagents, which can react with several functional groups. 

This paper introduced a novel derivatization-based strategy with 2-bromo-4′-nitroacetophenone (BNAP) after a simple protein precipitation step. The fact that BNAP is capable of reacting with several functional groups is very desirable because of the structural variability of Trp metabolites. Since derivatization is an extra step in sample preparation, optimization of the derivatization procedure was performed with respect to simplicity and practicability. The developed and validated RP-UHPLC-MS/MS method was used to simultaneously determine Trp and its metabolites from three different pathways related to neurodegenerative diseases in human plasma samples.

## 2. Materials and Methods

### 2.1. Chemicals and Reagents

Analytical standards of all analytes were purchased from Sigma Aldrich (Steinheim, Germany). Isotopically labeled internal standards (IS) were obtained as follows: PA-d4, NA-d4, and 3-IAA-d2 from Merck (Darmstadt, Germany); KA-d5 and Trp-indole-d5 from CDN isotopes (Pointe Claire, QC, Canada); XA-d4 from Santa Cruz Biotechnology (Dallas, TX, USA); 3-OH KYN-d3 from Buchem BV (Minden, Apeldoorn, Netherlands); QA-d3 and 5OH IAA-d6 from MedChemTronica (Sollentuna, Sweden); 3-OH AA-d3 and I3LA-d5 from LGC Standards (Łomianki, Poland); and AA-ring-13C6 from Eurisotope (Saint-Aubin, France). Sodium carbonate (Na_2_CO_3_), derivatization reagent (BNAP), ascorbic acid (AsA), and formic acid (FA) for LC-MS were obtained from Sigma Aldrich. LC-MS grade methanol (MeOH) and acetonitrile (ACN) were acquired from Honeywell (Seelze, Germany). Ultra-pure water (MPW) was made from distilled water using the Merck Millipore purification system from Merck.

### 2.2. Standards and Reagents Preparation

Stock standard solutions of all analytes were prepared in MPW:MeOH (1:1) + 0.1% FA + 0.1 µM AsA in concentration 1 mg/mL and were aliquoted and stored under −80 °C for further use. AsA was added into solutions to prevent the oxidation of analytes. Isotopically labeled internal standards were prepared in the same manner except for NA-d4, which was prepared in DMSO/MPW:MeOH (1:1) + 0.1% FA + 0.1 µM (50/50, *v*/*v*), and KA-d5, XA-d4, which were prepared in DMSO at a concentration of 1 mg/mL and was aliquoted and stored under -80 °C for further use. Standards and IS were then diluted with water as needed. Derivatization reagent (BNAP) was prepared fresh every day, right before the derivatization procedure, by dissolving the appropriate amount of BNAP in ACN (i.e., 200 mg of BNAP was dissolved in 5 mL of ACN).

### 2.3. Calibration Solutions

Stock solutions of individual analytes were appropriately diluted, mixed, and serially diluted to desired concentrations. All solutions were diluted with MPW. Calibration standards for PA, AA, 3-OH AA, KA, and XA were in the range of 1–100 ng/mL (individual concentrations were 1, 2.5, 5, 10, 25, 50, and 100 ng/mL); NA and 5-OH IAA were in the range of 5–500 ng/mL (individual concentrations were 5, 12.5, 25, 50, 125, 250, and 500 ng/mL); QA and I3LA were in the range of 10–1000 ng/mL (individual concentrations were 10, 25, 50, 100, 250, 500, and 1000 ng/mL); 3-OH KYN was in the range of 25–2500 ng/mL (individual concentrations were 25, 62.5, 125, 250, 625, 1250, and 2500 ng/mL); and Trp was in the range of 500–50,000 ng/mL (individual concentrations were 500, 1250, 2500, 5000, 12,500, 25,000, and 50,000 ng/mL). IS were prepared by dilution stock solutions with MPW and mixed to desired concentration levels. Concentrations of IS were constant in all calibration solutions as follows: 50 ng/mL for AA, 3-OH AA, and XA; 100 ng/mL for PA, NA, and KA; 1000 ng/mL for QA, I3LA, 5-OH IAA, and 3-OH KYN; and 5000 ng/mL for Trp. 

### 2.4. Sample Treatment and Derivatization Procedure

The derivatization procedure was optimized by a multistep optimization process. Optimized parameters were time (in the range of 0–3 h) and temperature (in the range of 20–90 °C) of the derivatization procedure, concentration (in the range of 1–80 mg/mL), and volume (in the range of 25–250 µL) of BNAP. Frozen (−80 °C) aliquots of the human plasma samples were thawed to laboratory temperature and immediately processed by the optimal procedure as follows: 50 µL of human plasma sample or calibration solutions in a 1.5 mL Eppendorf tube was mixed with 50 µL of MPW and 10 µL of IS mix. Then, 200 µL of ACN was added to precipitate proteins. The mixture was vortexed and centrifuged for 10 min at 10 °C and 30,000× *g*. The supernatant was transferred into a new tube and evaporated to dryness using the Eppendorf Concentrator plus. After that, the dried extract was resuspended in 20 µL of MPW, 20 µL of ACN, and 10 µL of 100 mM Na_2_CO_3_, vortexed, and rested for 5 min. Then, 150 µL of BNAP (40 mg/mL) was added, and the mixture was vortexed, stunned down, and allowed to react for 60 min at 50 °C in a dry bath incubator. After derivatization completion, the mixture was again vortexed, centrifuged for 10 min at 30,000 × *g* and 10 °C, and transferred into vials for UHPLC-MS/MS analysis.

All experiments with human plasma samples were approved by the Ethics Committee of St. Cyril and Methodius Hospital in Bratislava and the Ethics Committee of the Bratislava self-governing region.

### 2.5. UHPLC-MS/MS Analysis

All analyses were performed on an ACQUITY UPLC^®^ I class system (Waters, Milford, MA, USA) consisting of a binary solvent manager, sample manager flow-through needle (FTN), and column manager equipped with a reversed-phase column, Waters ACQUITY UPLC^®^ HSS T3 1.8 μm (2.1 × 100 mm) and ACQUITY UPLC^®^ HSS T3 1.8 μm (2.1 × 5 mm) VanGuard pre-column. The mobile phase consists of 0.1% FA in MPW (A) and ACN (B). For analyte separation, fast gradient elution, with total analysis time of 6 min, was used starting at 20% of B with linear increasing to 70% in 2 min (0–2 min), and then to 90% in 2 min (2–4 min), Next, hold for 0.5 min (4.0–4.5 min) and return to initial conditions in 0.5 min (4.5–5.0 min) and then perform a re-equilibration for 1 min (5.0–6.0 min). The flow rate of the mobile phase was 0.4 mL/min, the column temperature was 30 °C, and the sample injection volume was 5 µL. The LC system was connected with a triple quadrupole mass spectrometer, Xevo TQD (Waters, Milford, MA, USA), equipped with an electrospray ionization source (ESI) working in positive mode. ESI+ conditions were as follows: cone gas flow 50 L/h, desolvation gas flow 800 L/h, source temperature 150 °C, desolvation temperature 350 °C, and capillary voltage 3.5 kV. Collision energies and source cone voltage were manually tuned for each analyte and corresponding IS. The LC-MS system was controlled, and all data were acquired by MassLynx V4.2 software (Waters, Milford, MA, USA). Moreover, a total of four other columns with different chemistry were tested: ACQUITY UPLC^®^ CSH C18 1.7 μm (2.1 × 100 mm), ACQUITY UPLC^®^ CORTECS C18+ 1.6 μm (2.1 × 100 mm), Kinetex F5 1.7 μm (2.1 × 100 mm) (Phenomenex, Torrance, CA, USA), and ACQUITY UPLC^®^ HSS T3 1.8 μm (2.1 × 50 mm).

### 2.6. Method Validation

For method validation, quality control (QC) samples were prepared at four concentration levels: QC LLOQ (lower limit of quantification), QC low, QC medium, and QC high, from pooled human plasma samples. QC levels were prepared in concentrations according to the ICH M10 guideline [38] (the QC LLOQ level; QC low: within three times of LLOQ, QC medium: around 30–50% of the calibration curve range; QC high: at least 75% of the ULOQ (upper limit of quantification). Specific concentration levels of individual QC samples are shown in Table 1. The preparation of the QC samples for analysis was very similar to the previous procedure described in Section 2.4 except for one step, which included adding 50 µL corresponding QC level to the pooled human plasma sample instead of 50 µL of MPW. After that, all QC samples undergo the same derivatization step as described in Section 2.4. 

### 2.7. Data Analysis

All data obtained during the analyses were processed using different software, depending on the need: MassLynx 4.2 (Waters, Milford, MA, USA), Microsoft Excel 365 (Microsoft Corporation, Redmont, Birmingham, AL, USA), and GraphPad Prism 8.0.2 (GraphPad Software, San Diego, CA, USA). Differences between means were analyzed using an unpaired *t*-test. Differences at *p* < 0.05 were accepted as statistically significant.

## 3. Results and Discussion

### 3.1. Optimization of Derivatization

**Figure 2:**
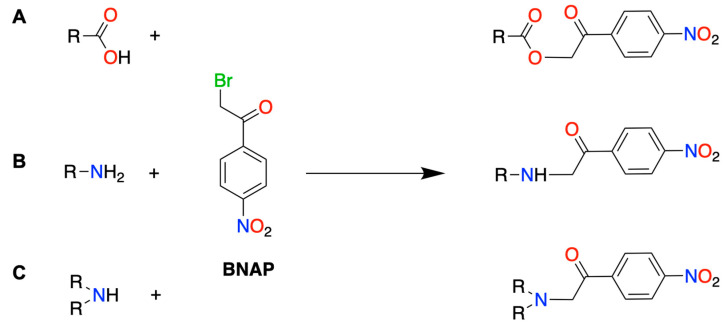
Illustration of derivatization reaction of BNAP with A: carboxyl; B: primary amine; and C: secondary amine group.

About 65% of the ~5000 known endogenous human metabolites contain at least one carboxylic acid group in their chemical structure [39]. Several acetophenone-based derivatization agents, such as 2-Bromo-1-phenylethanone, 1-(2,4-Dibromophenyl)ethenone [40], and 2-Bromo-1-[4-(dimethylamino)phenyl]ethenone [39], have been previously used for derivatization of carboxylic acids (CAs). To our best knowledge, BNAP was previously utilized only in the analysis of valproic acid in human plasma [41] or bile acids in *Calculus bovis* [42]. All of these derivatization reagents could easily react not only with carboxylic acid (R-COOH) but also with primary (R-NH_2_) and secondary (R-NH-R) amines (Figure 2). Moreover, BNAP can easily react with thiols (R-SH). This unique property (possibility of reaction with several functional groups) of reagents, like BNAP, gives the analytical process versatility. The biggest advantage of using these reagents is the possibility to derivatize a very wide range of analytes containing any of the above-mentioned functional groups in one run.

Optimization of the derivatization procedure was performed by a multistep optimization process by monitoring peak areas of all analytes. Firstly, it was necessary to choose a suitable environment in which the reaction between Trp metabolites and BNAP would take place. For the successful reaction course, an excess of organic solvent in the reaction (ACN) was necessary, as well as a basic environment provided by 100 mM Na_2_CO_3_. After that, temperature (*T*) was the second optimized parameter. Maximum conversion of analytes was achieved in the T range of 30–50 °C. Below this range, the derivatization reaction was incomplete, and above 60 °C, the resulting derivatives apparently were degraded. An example of the behavior of selected analytes at individual temperatures is shown in Figure S1A. Based on these data, 50 °C was chosen as the optimal temperature. In the optimization of reaction time (*t*), peak areas of AA, KA, XA, and QA were constantly increasing with time and reached a maximum after 3 h (the highest monitored point). Different behavior showed 3-OH AA, whose peak area started to decrease after one hour of derivatization (Figure S1B). Another optimized parameter was the concentration of BNAP (in the range of 1–80 mg/mL). A BNAP concentration of 40 mg/mL was chosen as optimal because lower concentrations of BNAP did not ensure the formation of a sufficient number of products for several analytes. Also, at a concentration of BNAP higher than 50 mg/mL, there was a significant decrease in the production of derivatives for several analytes. The dependence of peak area with BNAP concentration is shown in Figure S1C. The last optimized parameter was the volume of BNAP (in the range of 25–250 µL). The peak areas of almost all analytes increased with increasing BNAP volume. This is also due to the fact that BNAP was dissolved in ACN, which is necessary for the reaction because the reaction does not take place in an aqueous environment. However, in some cases, there was a reduction in product formation (as illustrated in Figure S1D). This phenomenon could probably be caused by reaching a state of saturation for some analytes (ideally, all molecules of analyte converted to the corresponding derivative) and the simultaneous increase of the total volume, which could cause dilution. Other steps of sample pretreatment, such as the selection of an appropriate precipitation solvent (methanol, trichloroacetic acid, ACN), volumes of all solvents used, or centrifugation parameters, were pre-optimized. 

In addition to these 11 analytes, I3AA, SER, and TRM were initially comprised in this method, and all of these analytes undergo the same derivatization step optimization. However, we were not able to determine TRM due to its low plasma concentration levels (under 1 ng/mL) and the low sensitivity of the method for TRM, and thus, this analyte was excluded from the method before validation. In the case of I3AA and SER, most likely due to the significant matrix effect observed in the method validation process, we excluded these analytes from the method due to repeated validation failure. Another possible reason for the unsuccessful validation of these analytes could be variations in the whole sample pretreatment step, such as evaporation after protein precipitation and/or derivatization process (derivatization yield, reaction kinetics), causing the issues with consistency of analytes responses and thus leading to validation failure.

### 3.2. RP-UHPLC-MS/MS Method Development

For optimization of MS detection, each analyte was derivatized separately. In the first step, an MS scan was used in the range of 50–800 *m*/*z* to cover the expected *m*/*z* values of derivatives. After determining the *m*/*z* of the precursor (parent) ions, the daughter mode was used to determine the characteristic fragmentation and to obtain the daughter (product) ions of each derivative. Subsequently, in order to achieve the highest possible sensitivity, parameters, such as cone voltage (in the range of 20–40 V) and collision energy (*C*e) (in the range of 10–40 eV), were tuned manually for each analyte. Based on the data obtained by these analyses, it was possible to create the final MRM method. Characteristics of the MRM method are listed in Table 2.

As can be seen in Table 2, there was no specific common fragment (product ion) for all analytes derived from the derivatization agent, as can be seen, i.e., in the case of benzoyl chlorid-derivatized analytes [43]. In our case, each analyte had specific fragments (product ions) derived based on the structure of the original molecule.

**Figure 3:**
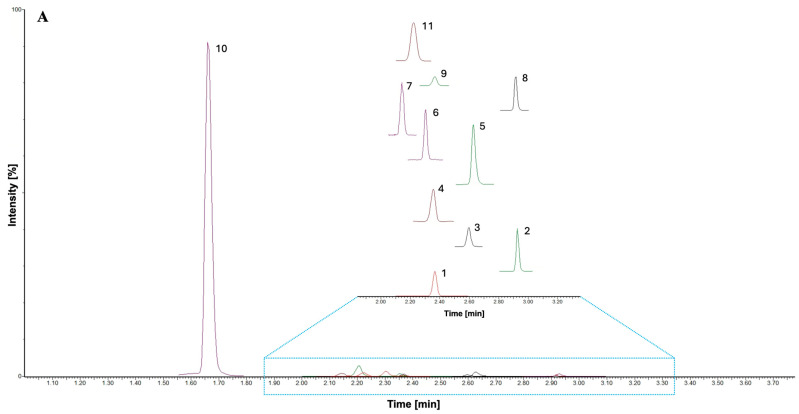

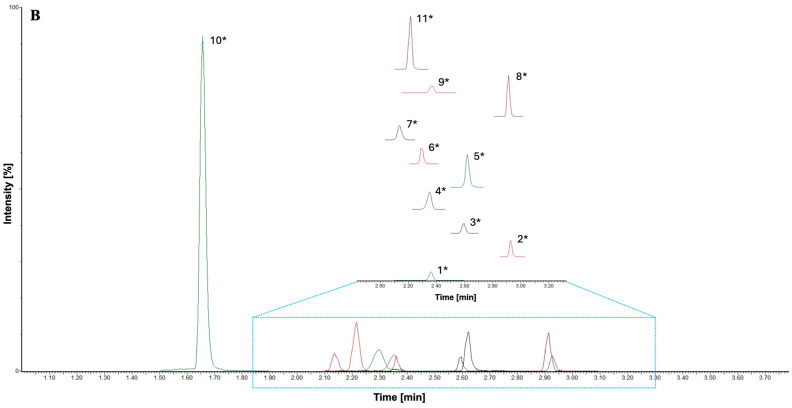
Representative MRM chromatograms of (**A**) BNAP derivatives and (**B**) corresponding IS (tagged with *): 1: 3-OH KYN; 2: AA; 3: 3-OH AA; 4: 5-OH IAA; 5: I3LA; 6: PA; 7: XA; 8: QA; 9: NA; 10: Trp; 11: KA.

Due to the development of a new method, in addition to the selection of a suitable stationary phase, it was necessary to optimize all chromatographic parameters, such as the composition and flow rate of the mobile phase, column temperature, and sample injection. Among the tested columns, the ACQUITY UPLC^®^ HSS T3 1.8 μm (2.1 × 100 mm) column proved to be the most suitable for sufficient separation of BNAP derivatives and satisfactory peak shapes. The tested column temperatures (30, 35, and 40 °C) did not show significant changes, but the best peak shapes were achieved at a column temperature of 30 °C. The sample injection was set to 5 µL among the tested injection volumes (1, 5, and 10 µL). To prevent the carryover effect, 50% ACN was used to rinse the injection needle. To ensure the lowest possible carryover, the needle wash before and after injection was set to 3 and 6 sec, respectively. As part of method development, different compositions of the mobile phase (0.1% FA in MPW, 0.1% FA in MPW, pH 3.0, or 0.5% FA in MPW) were tested. After visual evaluation of the chromatograms, 0.1% FA in MPW (A) with pH 2.5 and ACN (B) was chosen. The optimization of the mobile phase, column temperature, and choice of the suitable stationary phase was evaluated in terms of peak symmetry, expressed as a tailing factor (*T*_F_) (Table S1). Injection volume optimization was evaluated by visual comparison of UHPLC-MS/MS chromatograms for three representative derivatives 5-OH IAA, I3LA, and QA (Figure S2). The next phase of method development was to set up gradient elution. All analyte derivatives eluted between 1.67 min and 2.94 min and in 61–80% of B. The use of the multiple reaction monitoring (MRM) mode together with IS enabled the reliable identification and quantification of analytes that were not chromatographically separated, i.e., QA and AA. However, the developed method was able to separate a pair of positional isomers (PA, NA) with the *t*_R_ 2.31 min and 2.38 min, respectively. A typical MRM chromatogram of BNAP derivatives separated by the developed RP-UHPLC-MS/MS method is shown in Figure 3.

### 3.3. UHPLC-MS/MS Method Validation

The developed method was validated in terms of linearity, sensitivity (LLOQ), limit of detection (LOD), stability, accuracy, and precision. The validation procedure was performed according to the ICH guidelines for bioanalytical method validation [38]. A summary of the validation results is shown in Table 3, Table 4, Table 5 and Table 6. Concentrations of calibration solutions have been chosen in the aforementioned ranges, according to the literature, to cover the expected concentrations of analytes in human plasma. The linearity of the method was proven by measuring the calibration solutions of all analytes in the concentration ranges set, as shown in Table 3. The method showed very good linearity; *R*^2^ was higher than 0.99 for all analytes in selected concentration ranges. Theoretical LODs for all analytes were calculated from calibration curves (except Trp). Due to the high LLOQ concentration of Trp, we were not able to calculate the LOD correctly. Thus, for this purpose, a calibration curve in a low concentration area (1–25 ng/mL) for Trp was prepared, and the LOD was then calculated from this calibration curve. LODs for all analytes were in the range of 0.37–10.97 ng/mL. The method proves to be sensitive enough to detect physiological concentrations of analytes in human plasma samples. The lowest point of each calibration curve represents the LLOQ values. The highest calibration point was set as ULOQ. Another investigated parameter for method validation was carryover. According to the ICH guidelines, carryover should not exceed 20% response of LLOQ. In all cases, no carryover (max. 8.8%) was observed in the blank after injection of ULOQ standard solution (the highest point of the calibration curve) (Table 3).

Accuracy and precision were calculated from the analysis of QC samples. QC samples were prepared at four concentration levels: LLOQ, low, medium, and high from pooled human plasma. Accuracy (expressed as % comparing nominal and found concentration of each analyte) and precision (expressed as RSD %) were determined by analyzing five replicates of each QC level in one day (intra-day) and over three days (inter-day). The developed method was evaluated as precise and accurate. All values of accuracy and precision have met the ICH M10 acceptance criteria, as accuracy values were in the range of 93.3–110.1%, and RSD values were, in all cases, less than 9% (Table 4). Evaluation of recovery is very important in the analysis of biological samples, especially in the analysis of endogenous compounds. To determine the matrix effect, total recovery was performed as part of the method validation. Recovery was calculated by comparing the analyte response obtained from analyzing QC plasma samples to the analyte response obtained from analyzing standard samples, which were prepared in LC-MS grade water at equivalent concentration levels. Recovery values of individual analytes are shown in Table 4. There was no significant influence of the matrix on the monitored analytes was observed.

Two types of stability measurements relevant to our study were conducted: autosampler stability and freeze–thaw stability. Firstly, the stability of analytes in human plasma samples was determined after storing them for 24 h in a UHPLC autosampler with a temperature set at 10 °C. Freeze–thaw stability was evaluated after the completion of three freeze–thaw cycles (15 h between each cycle). In both types of stability, the accuracy of all monitored analytes was in the range of 90.1–112.0%, which is within the acceptance criteria of the ICH M10 guideline. A summary of stability evaluation is presented in Table 5.

Robustness indicates whether the proposed method is able to provide equally reliable results, even with a small change in analysis conditions. For robustness assessment, all QC levels were analyzed under “normal” (optimal) conditions and under deliberately changed conditions, such as mobile phase flow rate (± 0.01 mL/min), column temperature (± 1 °C), and % of B mobile phase (ACN) at the beginning of gradient elution (± 1%). The results of the responses were then directly compared with those obtained by the analysis of the same QC samples under “normal” conditions and are expressed as RSD. The overview of robustness evaluation is summarized in Table 6.

According to the data, the method was robust under these small changes in separation conditions. In all cases, RSD was less than 9%. Robustness does not have acceptance criteria, but generally, a method is considered robust if the bias is less than 10% and repeatability is less than 25% [44]. Change in mobile phase flow rate (± 0.01 mL/min) and % of B mobile phase at the beginning of gradient elution (± 1%) caused a small shift in retention times. However, it had no negative impact on separation or analyte responses. 

**Figure 4:**
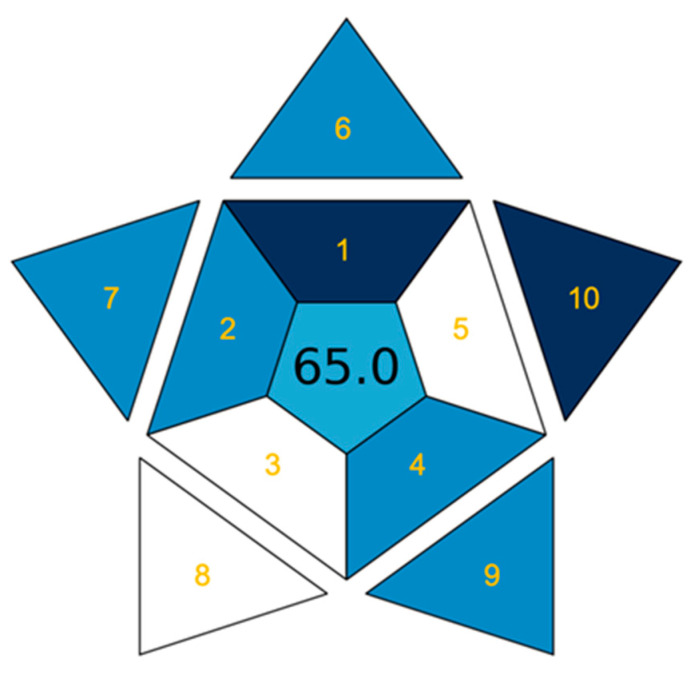
Evaluation of our developed RP-UHPLC-MS/MS method for Trp and its 10 metabolites using the BAGI metric tool.

Finally, a recently introduced metric tool, the blue applicability grade index (BAGI), published by Manousi et al. [45], was used to evaluate method practicability. The BAGI is based on 10 criteria (see Figure 4) comprised in the analytical procedure. For each criterion, 2.5, 5.0, 7.5, or 10.0 points with the corresponding color (white, light blue, blue, and dark blue) could be assessed. The BAGI assessment results in an asteroid pictogram shaded with resulting colors accompanied by the total score of method evaluation. Generally, the method is considered practical when the BAGI score is at least 60 points. The BAGI assessment of our developed RP-UHPLC-MS/MS method with a score of 65 points with the corresponding pictogram is shown in Figure 4. On the other hand, according to the final score, our method can be considered practical and suitable for bioanalytical applications.

### 3.4. Quantification of Trp Metabolites in Human Plasma Samples

After validation, the RP-UHPLC-MS/MS applicability of the method was proven using the determination of Trp and its 10 metabolites in human plasma samples. A total of 60 plasma samples were analyzed, of which 30 were plasma from patients suffering from AD and 30 samples were healthy controls (CTRLs). All aliquots of human plasma samples were frozen at −80 °C. Before the sample treatment procedure, human plasma samples were thawed to laboratory temperature and immediately processed by simple protein precipitation, supernatant evaporation, and a derivatization procedure, as described in Section 2.4. Basic demographic information of the subjects is shown in Table 7. 

**Figure 5:**
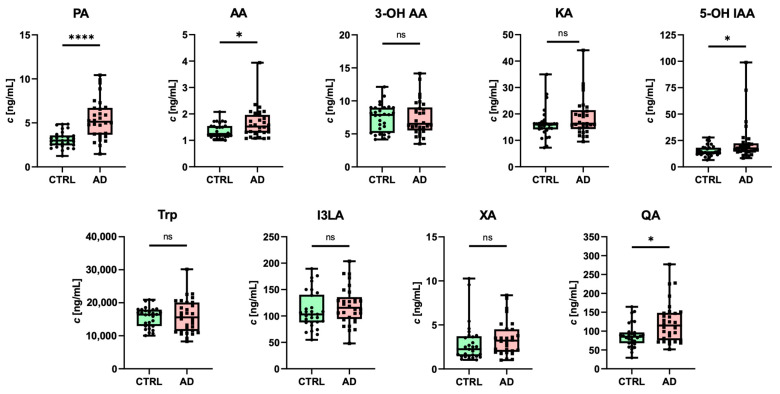
Quantification of Trp metabolites in human plasma samples from AD patients and age-matched CTRLs: * *p* < 0.05; **** *p* < 0.0001; ns—nonsignificant.

Despite using derivatization to increase the sensitivity of the method, concentrations of 3-OH KYN and NA were below their LLOQ in most samples, and thus, we were not able to quantify them with sufficient analytical reliability. All other analytes were reliably detected with the developed RP-UHPLC-MS/MS method. Changes in monitored analyte concentrations in human plasma samples from the CTRL and AD patients were statistically evaluated (Figure 5).

Several analytes showed sex-specific differences between the CTRL and AD groups. We detected significant differences in PA (both sexes), AA (females), KA (females), 5-OH IAA (females), XA (males), I3LA (females), and QA (females) in comparison to the CTRL and AD groups (Table 8, Figure S3). 

Alterations in some KP metabolites can be associated with the activation of KP in various pro-inflammatory conditions [46]. Elevated levels of neurotoxic QA in plasma are consistent with the study published by Gulaj et al. [47]. Moreover, the same study [47] reported elevated levels of AA in AD patients. Interestingly, it has been shown that higher plasmatic concentrations of AA can be assigned to a greater future risk of dementia and AD [46]. Surprisingly, our results show elevated levels of neuroprotective PA in both groups of AD patients. In AD patients, plasma levels of PA were previously shown to have a negative correlation with CSF total tau, suggesting that PA may play a protective role against tau pathology [48]. Further studies are needed to understand better the role of PA in the development of tau pathology.

In AD females, KA was significantly elevated. This indicates that a portion of KYN is metabolized toward neuroprotective KA. Consistent with [49], we observed that KA levels were higher in healthy males in comparison to healthy females, suggesting the effect of circulating sex steroid hormones on the immune-regulating kynurenine pathway [50]. The levels of XA were only significantly elevated in AD males, and no differences were found in females. This is in contrast to a previous study where XA plasma levels decrease with the progression of the disease [51]. Alterations in the main SER metabolites, 5-OH IAA and I3LA, were observed in our study. Increased levels of 5-OH IAA were observed mainly in autism spectrum disorders [52]. To our knowledge, only one recent study was dedicated to the association of 5-OH IAA in AD [14]. However, only CSF alterations of 5-OH IAA were examined in this study. 

To our best knowledge, there are only three recently published papers (one protocol included) dealing with the derivatization of Trp and its metabolites. In a paper by Tömösi et al. [35], the authors used esterification to derivatize QA, PA, and 3-OH KYN. However, the authors only used derivatization to improve chromatographic properties in the RP-LC system. Vondroušová et al. [53] determined several kynurenine pathway metabolites, neurotransmitters, and their metabolites after their derivatization with 3-aminopyridyl-*N*-hydroxysuccinimidyl carbamate (APDS) (for -NH_2_ group derivatization) and 2-aminoethyl trimethyl ammonium chloride, *N*-hydroxysulfosuccinimide (sulfo-NHS) (for -COOH group derivatization). Thanks to this procedure, the authors increased the response of the MS detection and analyte stability. Another study (protocol) conducted on Trp and its metabolites comprising derivatization with *o*-phthaldialdehyde (OPA) prior to their LC-FLD determination was performed by Dai et al. [54]. The method showed good potential for the determination of Trp and its metabolites in biological samples.

This study is not exempt from limitations. First, our method was not sensitive enough to analyze 3-OH KYN and NA, two important metabolites of the KYN pathway. Second, some discrepancies in concentration changes of Trp and its metabolites with other studies, i.e., [55], can be caused by high variability of biological samples and also factors affecting metabolite levels, such as smoking or other associated diseases that we did not include in the analysis. To reliably reveal differences in concentrations of metabolites, it is essential to constantly improve and develop new methods for sensitive determination of as many analytes as possible in one run.

## 4. Conclusions

Despite advancements in the instrumentation of LC and mass spectrometry, analytical challenges related to the physicochemical characteristics of monitored analytes, such as inadequate polarity for effective separation, limited ionization efficiency in MS, or the absence of chromophores for UV detection, persist. Consequently, sensitivity may be compromised. These challenges can be effectively addressed through derivatization techniques. In this study, we utilized an acetophenone-based derivatization reagent, BNAP, for the chemical modification of Trp and its ten important metabolites prior to their LC-MS analysis, which was validated according to the ICH guidelines. The values of all validation parameters met the acceptance criteria. Finally, method practicability was evaluated using the BAGI metric system; with a score of 65, the method can be considered practical. Method applicability was confirmed by analysis of human plasma samples from patients with Alzheimer’s disease and healthy controls, respectively. Analysis reveals significant differences in several metabolites. BNAP showed great potential in labeling analytes with different functional groups, resulting in the possibility to analyze many different analytes in one run.

## Figures and Tables

**Table 1:** Concentration levels of individual QC samples.

Analyte	QC LLOQ [ng/mL]	QC Low [ng/mL]	QC Medium [ng/mL]	QC High [ng/mL]
PA	1	2	40	80
AA				
3-OH AA				
KA				
XA				
NA	5	10	200	400
5-OH IAA				
QA	10	20	400	800
I3LA				
3-OH KYN	25	50	1000	2000
Trp	500	1000	20,000	40,000

**Table 2:** The overview of selected MRM transitions, cone voltages, and collision energies for individual BNAP derivatives and their IS.

Analyte	Precursor Ion	Product Ion (Qualitative) *	Product Ion (Quantitative)	Cone [V]	Ce [eV]
PA	287	106	78	35	20
PA-d4	291	110	82	35	20
NA	287	90	241	30	30
NA-d4	291	-	245	30	30
AA	301	92	120	25	15
AA-ring-13C6	307	-	126	25	15
3-OH AA	317	108	136	20	25
3-OH AA-d3	320	-	139	20	20
KA	353	172	144	30	20
KA-d5	358	177	149	30	20
XA	369	188	160	30	25
XA-d4	373	192	164	30	25
QA	494	164	313	35	15
QA-d3	497	-	316	30	20
Trp	368	159	170	25	25
Trp-indole-d5	373	-	164	25	25
3-OH KYN	551	190	152	30	30
3-OH KYN-d3	554	-	155	30	20
I3LA	369	170	118	25	35
I3LA-d5	374	-	123	25	30
5-OH IAA	355	118	146	30	30
5-OH IAA-d6	361	-	152	30	25

* Some IS had only one product ion (fragment) in the MS/MS spectrum, which was used for reliable identification and quantification. This phenomenon is common in the case of IS due to the incorporation of a stable isotope to the molecule of analyte, which can cause alterations in fragmentation or mass shift.

**Table 3:** Overview of selected basic operational and validation parameters.

Analyte	*t*_R_ [min]	Linear Range [ng/mL]	*R* ^2^	LOD [ng/mL]	LLOQ [ng/mL]	Carryover [%]
PA	2.31	1–100	0.9978	0.40	1	8.8
NA	2.38	5–500	0.9958	2.43	5	0.8
AA	2.94	1–100	0.9980	0.25	1	6.0
3-OH AA	2.61	1–100	0.9947	0.15	1	0.2
KA	2.23	1–100	0.9957	0.60	1	2.8
XA	2.15	1–100	0.9966	0.57	1	0.4
QA	2.92	10–1000	0.9961	4.50	10	1.9
Trp	1.67	500–50,000	0.9994	0.86 *	500	2.4
3-OH KYN	2.38	25–2500	0.9942	9.43	25	0.7
I3LA	2.64	10–1000	0.9955	7.81	10	0.4
5-OH IAA	2.36	5–500	0.9982	1.33	5	0.3

* LOD value for Trp calculated from an extra calibration curve in a low concentration area (1–25 ng/mL).

**Table 4:** Results of intra- and inter-day accuracy, precision, and recovery of the developed RP-UHPLC-MS/MS method.

			Intra-Day (*n* = 5)			Inter-Day (*n* = 15)			*n* = 6
Analyte	QC Level	Nominal *c* [ng/mL]	Found [ng/mL]	Accuracy [%]	RSD [%]	Found [ng/mL]	Accuracy [%]	RSD [%]	Recovery [%]
PA	LLOQ	1	1.00	100.4	6.6	1.00	100.2	5.5	90.4
	Low	2	2.14	106.6	4.8	2.10	105.2	5.9	100.0
	Medium	40	40.48	101.2	2.9	40.04	100.1	3.2	97.2
	High	80	79.68	99.6	3.0	79.71	99.6	3.1	103.6
NA	LLOQ	5	5.96	97.6	5.9	4.90	98.1	5.3	90.7
	Low	10	10.87	97.9	8.2	9.89	98.9	7.2	103.7
	Medium	200	200.33	99.6	2.1	196.87	98.4	5.0	97.6
	High	400	393.93	98.2	4.3	385.98	96.5	3.1	98.7
AA	LLOQ	1	1.01	101.0	6.5	1.01	101.1	5.3	92.7
	Low	2	2.00	100.2	4.2	2.00	100.4	4.6	109.0
	Medium	40	38.88	97.2	3.4	39.82	99.6	3.8	98.6
	High	80	78.61	98.3	5.4	79.61	99.5	4.1	101.3
3-OH AA	LLOQ	1	0.98	97.9	4.9	0.98	97.7	6.7	83.3
	Low	2	2.03	101.6	6.7	2.03	101.6	5.8	94.6
	Medium	40	39.72	99.3	4.8	40.67	101.7	3.2	97.8
	High	80	81.40	101.7	4.4	82.18	102.7	4.1	97.8
KA	LLOQ	1	1.03	103.0	7.0	1.03	102.7	6.0	100.8
	Low	2	2.08	104.2	6.8	2.03	101.5	5.6	89.5
	Medium	40	40.24	100.6	2.8	42.03	105.1	2.3	105.2
	High	80	79.13	98.9	6.5	83.66	104.6	5.7	102.7
XA	LLOQ	1	1.01	100.5	4.7	1.02	101.7	6.4	88.1
	Low	2	2.19	109.4	3.0	2.12	106.0	4.7	80.5
	Medium	40	40.02	100.0	4.7	39.28	98.2	4.8	85.4
	High	80	83.35	104.2	2.2	80.45	100.6	2.9	88.0
QA	LLOQ	10	9.99	99.9	4.5	10.04	100.4	5.6	85.1
	Low	20	19.98	99.9	3.5	20.36	101.8	5.3	84.0
	Medium	400	422.78	105.7	6.6	409.44	102.4	4.4	95.4
	High	800	807.93	101.0	4.3	805.38	100.7	5.2	97.3
Trp	LLOQ	500	503.24	100.6	1.4	498.16	99.6	4.9	95.5
	Low	1000	1008.51	100.9	4.7	1003.68	100.4	5.3	80.6
	Medium	20,000	19,826.20	99.1	6.4	19,853.62	99.3	5.7	90.2
	High	40,000	39,031.26	97.6	6.0	39,083.29	97.7	4.7	88.4
3-OH KYN	LLOQ	25	26.44	105.8	0.5	26.75	107.0	3.2	104.4
	Low	50	54.67	109.3	6.5	54.04	108.1	5.6	98.2
	Medium	1000	1096.64	109.7	3.3	1095.45	109.6	4.5	96.9
	High	2000	2179.48	109.0	4.0	2201.95	110.1	4.4	98.7
I3LA	LLOQ	10	10.17	101.7	7.3	10.11	101.1	6.4	87.3
	Low	20	20.70	103.5	7.1	20.35	101.7	6.1	87.4
	Medium	400	381.10	95.3	5.8	378.80	94.7	7.4	83.4
	High	800	759.23	94.9	3.3	764.41	95.5	3.9	80.7
5-OH IAA	LLOQ	5	5.12	102.5	7.0	5.096	101.9	5.4	98.7
	Low	10	10.00	99.9	2.1	10.00	100.0	3.7	96.9
	Medium	200	186.59	93.3	6.5	195.08	97.5	5.6	99.8
	High	400	395.50	98.9	2.5	399.55	99.9	3.4	98.2

**Table 5:** Summary of stability assessment of Trp metabolite derivatives.

			Autosampler (*n* = 5)			Freeze–Thaw (*n* = 5)		
Analyte	QC Level	Nominal *c* [ng/mL]	Found [ng/mL]	Accuracy [%]	RSD [%]	Found [ng/mL]	Accuracy [%]	RSD [%]
PA	LLOQ	1	1.00	100.2	8.6	1.04	101.1	4.9
	Low	2	2.05	102.4	9.8	2.17	108.6	1.0
	Medium	40	39.45	98.6	4.4	38.91	97.3	6.5
	High	80	79.07	98.8	0.6	79.55	99.4	1.4
NA	LLOQ	5	5.11	102.3	10.2	5.19	103.9	14.3
	Low	10	10.20	102.0	4.0	10.00	100.0	8.1
	Medium	200	207.19	103.6	3.8	188.04	94.0	8.1
	High	400	408.91	102.2	3.5	390.74	97.7	5.7
AA	LLOQ	1	1.02	101.6	3.5	0.97	97.2	14.3
	Low	2	2.03	101.2	3.3	1.92	95.9	8.6
	Medium	40	40.74	101.8	1.6	41.51	103.8	4.2
	High	80	80.62	100.8	3.3	85.85	107.3	7.5
3-OH AA	LLOQ	1	1.01	100.5	14.4	0.91	90.8	6.5
	Low	2	1.99	99.4	6.8	2.05	102.3	9.7
	Medium	40	39.40	98.5	2.0	40.54	101.3	3.5
	High	80	80.58	100.7	2.8	81.43	101.8	4.0
KA	LLOQ	1	1.01	101.2	7.3	0.95	95.2	9.8
	Low	2	1.99	99.3	8.0	2.10	105.1	10.4
	Medium	40	42.21	105.5	1.5	41.70	104.2	7.8
	High	80	81.58	102.0	1.8	84.70	105.9	7.8
XA	LLOQ	1	0.99	99.0	7.7	1.014	101.4	12.4
	Low	2	2.033	101.6	8.1	1.897	94.9	6.3
	Medium	40	39.44	98.6	4.6	38.24	95.6	5.5
	High	80	78.24	97.8	2.4	77.71	97.1	2.5
QA	LLOQ	10	10.17	101.7	5.9	10.16	101.6	9.4
	Low	20	19.93	99.7	10.6	21.15	105.8	4.7
	Medium	400	405.13	101.3	2.4	395.47	98.9	3.8
	High	800	797.64	99.7	8.8	817.14	102.1	8.9
Trp	LLOQ	500	506.88	101.4	7.5	492.62	98.5	14.2
	Low	1000	1013.99	101.4	4.7	955.40	95.5	8.3
	Medium	20,000	20,438.98	102.2	2.5	19,603.80	98.0	7.4
	High	40,000	39,922.96	99.8	2.5	40,087.04	100.2	4.9
3-OH KYN	LLOQ	25	24.42	97.4	6.6	27.58	110.3	12.3
	Low	50	50.70	101.4	3.4	53.02	106.0	6.0
	Medium	1000	1039.83	104.0	4.3	1033.51	103.4	3.7
	High	2000	2138.14	106.9	45.7	2239.98	112.0	5.5
I3LA	LLOQ	10	10.38	103.7	7.0	9.57	95.7	12.0
	Low	20	20.27	101.4	11.6	20.66	103.3	6.7
	Medium	400	405.01	101.3	1.3	429.28	107.3	4.6
	High	800	785.55	98.2	4.2	815.93	102.0	4.8
5-OH IAA	LLOQ	5	4.95	99.0	6.3	4.47	94.8	6.3
	Low	10	9.02	90.4	6.6	10.98	109.8	11.8
	Medium	200	188.79	94.4	5.8	180.13	90.1	5.2
	High	400	388.75	97.2	14.0	373.21	93.3	8.9

**Table 6:** Overview of RP-UHPLC-MS/MS method robustness.

Analyte	QC Level	*T*_C_ RSD [%]	*F*_R_ RSD [%]	% of B RSD [%]
PA	LLOQ	4.9	4.4	8.7
	Low	4.1	5.2	4.9
	Medium	3.6	5.6	4.9
	High	4.6	1.8	4.5
NA	LLOQ	3.3	6.0	6.2
	Low	7.0	5.5	1.0
	Medium	7.8	4.8	6.3
	High	1.7	4.4	4.7
AA	LLOQ	1.8	3.6	6.5
	Low	4.2	1.8	1.4
	Medium	3.3	2.7	3.0
	High	3.0	1.7	3.4
3-OH AA	LLOQ	7.9	1.5	6.9
	Low	4.1	5.4	7.5
	Medium	5.8	8.2	7.1
	High	4.2	7.8	5.3
KA	LLOQ	1.3	5.1	3.4
	Low	4.8	1.3	6.3
	Medium	5.0	5.8	4.3
	High	4.4	3.7	7.4
XA	LLOQ	4.6	4.5	8.1
	Low	7.7	5.5	4.7
	Medium	8.4	7.4	2.4
	High	8.9	5.9	7.0
QA	LLOQ	3.5	1.3	4.0
	Low	3.3	6.0	1.6
	Medium	6.2	9.1	4.6
	High	2.7	2.3	5.7
Trp	LLOQ	3.9	2.5	4.1
	Low	6.3	1.8	4.9
	Medium	2.5	6.4	4.8
	High	4.3	6.5	4.2
3-OH KYN	LLOQ	6.4	8.0	5.9
	Low	7.3	4.7	6.8
	Medium	2.3	7.9	5.6
	High	2.2	5.1	7.4
I3LA	LLOQ	2.9	5.5	1.9
	Low	4.1	4.5	7.3
	Medium	6.0	8.0	2.3
	High	5.7	2.4	2.6
5-OH IAA	LLOQ	5.5	2.8	1.1
	Low	2.0	2.0	3.8
	Medium	6.3	5.3	3.0
	High	5.9	8.8	8.9

*T*_C_: column temperature; *F*_R_: mobile phase flow rate.

**Table 7:** Basic demographic information of the subjects.

Group	CTRL	AD
Number of individuals (n)	30	30
Age (mean ± SD)	78.5 ± 4.3	82.6 ± 4.7
Sex (male/female)	10/20	11/19
MMSE (mean ± SD)	-	19.9 ± 7.6 *
MoCA (mean ± SD)	-	16.7 ± 5.8 *

* Nine patients were subjected to the Mini Mental State Exam (MMSE) and twenty-one patients were subjected to the Montreal Cognitive Assessment (MoCA).

**Table 8:** Sex-specific differences of Trp and its eight metabolites levels in plasma from CTRL and AD patients; *p*-values in bold were considered significant.

Analyte	Sex	Concentration (Mean ± SD) [ng/mL]		*p*-Value
		CTRL	AD	
PA	F	2.80 ± 0.74	5.11 ± 2.27	**0.0002**
	M	3.70 ± 0.89	5.96 ± 2.04	**0.0062**
AA	F	1.36 ± 0.30	1.66 ± 0.63	0.0790
	M	1.53 ± 0.45	1.61 ± 0.44	0.6987
3-OH AA	F	7.38 ± 1.95	7.25 ± 2.87	0.8715
	M	7.40 ± 2.20	7.85 ± 1.83	0.6301
KA	F	14.42 ± 3.16	19.36 ± 7.81	**0.0151**
	M	20.23 ± 6.76	17.87 ± 5.69	0.4198
5-OH IAA	F	13.80 ± 3.35	23.32 ± 19.23	**0.0405**
	M	18.46 ± 6.11	22.86 ± 17.48	0.4818
Trp	F	15,773.02 ± 2620.40	17,172.81 ± 4902.50	0.2825
	M	15,378.28 ± 3649.57	13,703.87 ± 4202.85	0.3676
I3LA	F	90.94 ± 16.76	114.75 ± 37.01	**0.0154**
	M	149.24 ± 27.78	127.07 ± 29.65	0.1097
XA	F	2.83 ± 1.97	2.34 ± 1.30	0.7063
	M	3.07 ± 1.21	4.95 ± 2.09	**0.0289**
QA	F	87.60 ± 32.72	127.79 ± 56.37	**0.0112**
	M	87.98 ± 27.40	114.02 ± 45.39	0.1513

## Data Availability

All data are available from the corresponding author upon request.

## Supplementary Materials

The following supporting information can be downloaded at https://www.mdpi.com/article/10.3390/biomedicines12051003/s1, Figure S1: Illustration of BNAP derivatization optimization of A: temperature; B: time; C: concentration of BNAP; and D: volume of BNAP; Table S1: Choice of a suitable stationary phase and the optimization of a mobile phase and column temperature evaluation based on tailing factors (*T*_F_) of three representative derivatives: 5-OH IAA, I3LA, and QA; *T*_F_ is presented as the mean value of three measurements; Figure S2: UHPLC-MS/MS chromatograms (without peak smoothing) of injection volume optimization for three representative derivatives: 5-OH IAA, I3LA, and QA; A: 1 µL; B: 5 µL; C: 10 µL; Figure S3: Statistical evaluation of quantification of Trp metabolites in female (A) and male (B) plasma samples from AD patients and age-matched CTRLs; * *p* < 0.05; ** *p* < 0.01; *** *p* < 0.01; ns—nonsignificant.
