# Supplementary material for: A Novel RP-UHPLC-MS/MS Approach for the Determination of Tryptophan Metabolites Derivatized with 2-Bromo-4′-Nitroacetophenone
Source: Biomedicines. 2024 May 2;12(5):1003. doi: 10.3390/biomedicines12051003 (PMC11117999; doi:10.3390/biomedicines12051003)

## Supplementary material

**Figure S1** Illustration of BNAP derivatization optimization of **A**: temperature; **B**: time; **C**: concentration of BNAP and **D**: volume of BNAP.

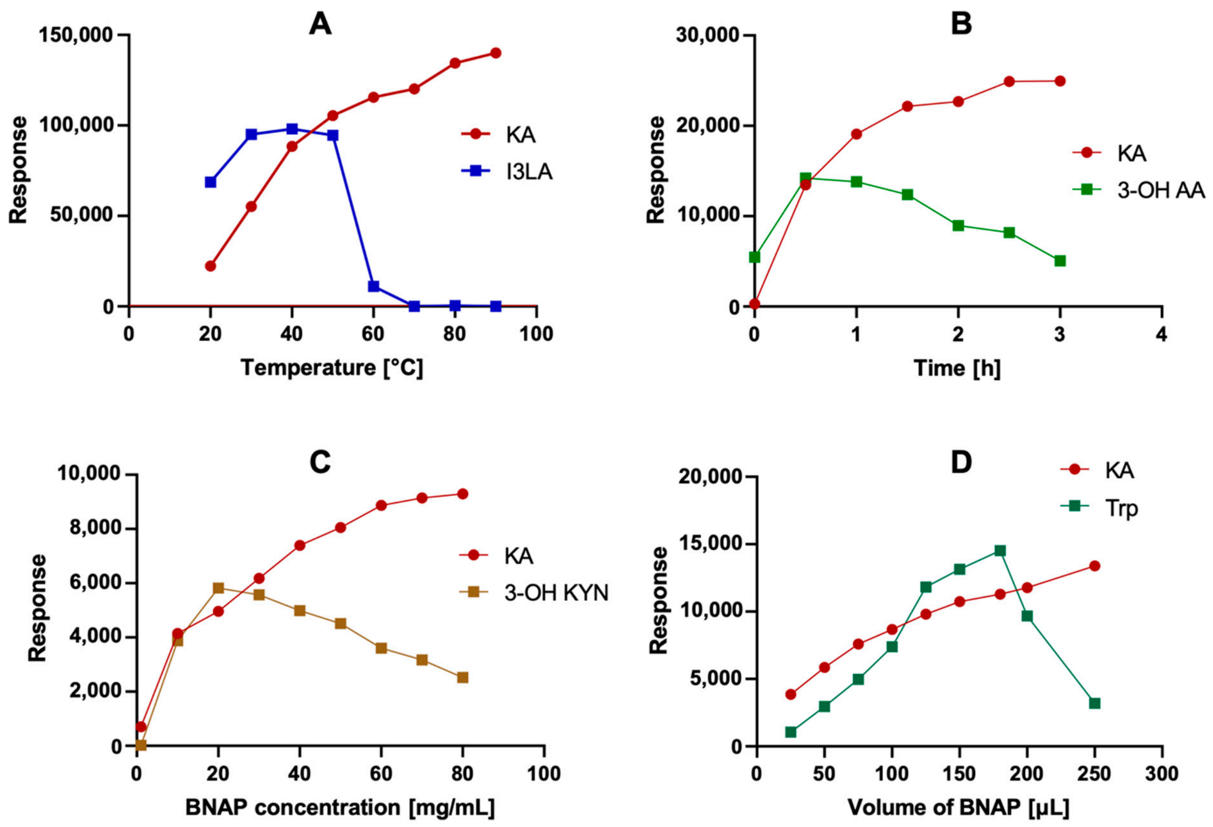

**Table S1** Chose of suitable stationary phase and optimization of mobile phase and column temperature evaluation based on tailing factors ($T_F$) of three representative derivatives 5-OH IAA, I3LA and QA. $T_F$ are presented as mean value of three measurements.

| **Column** | **Tailing factor ($T_F$)** | | |
|---|---|---|---|
| | **5-OH IAA** | **I3LA** | **QA** |
| HSS T3 (2.1×100 mm) | 1.06 | 1.85 | 1.15 |
| CSH C18 | 0.69 | 1.49 | 1.75 |
| CORTECS C18+ | 0.76 | 0.93 | 1.33 |
| Kinetex F5 | 1.58 | 1.58 | peak splitting |
| HSS T3 (2.1×50 mm) | 1.10 | 0.81 | 1.58 |
| **Mobile phase** | **Tailing factor ($T_F$)** | | |
| | **5-OH IAA** | **I3LA** | **QA** |
| 0.1% FA (pH 2.5) | 1.06 | 1.62 | 1.15 |
| 0.1% FA (pH 3) | 0.88 | 1.70 | 1.42 |
| 0.1% FA (pH 4) | 0.94 | 1.93 | 1.25 |
| 0.15 FA + 10 mM AF (pH 3) | 0.82 | 1.69 | 1.50 |
| 0.5% FA (pH 2.2) | 1.00 | 2.10 | 1.50 |
| **Column temperature [°C]** | **Tailing factor ($T_F$)** | | |
| | **5-OH IAA** | **I3LA** | **QA** |
| 30 | 1.06 | 1.85 | 1.10 |
| 35 | 0.98 | 2.44 | 1.10 |
| 40 | 1.23 | 2.12 | 1.69 |

**Figure S2** UHPLC-MS/MS chromatograms (without peak smoothing) of injection volume optimization for three representative derivatives 5-OH IAA, I3LA and QA; **A**: 1 μL; **B**: 5 μL; **C**: 10 μL.

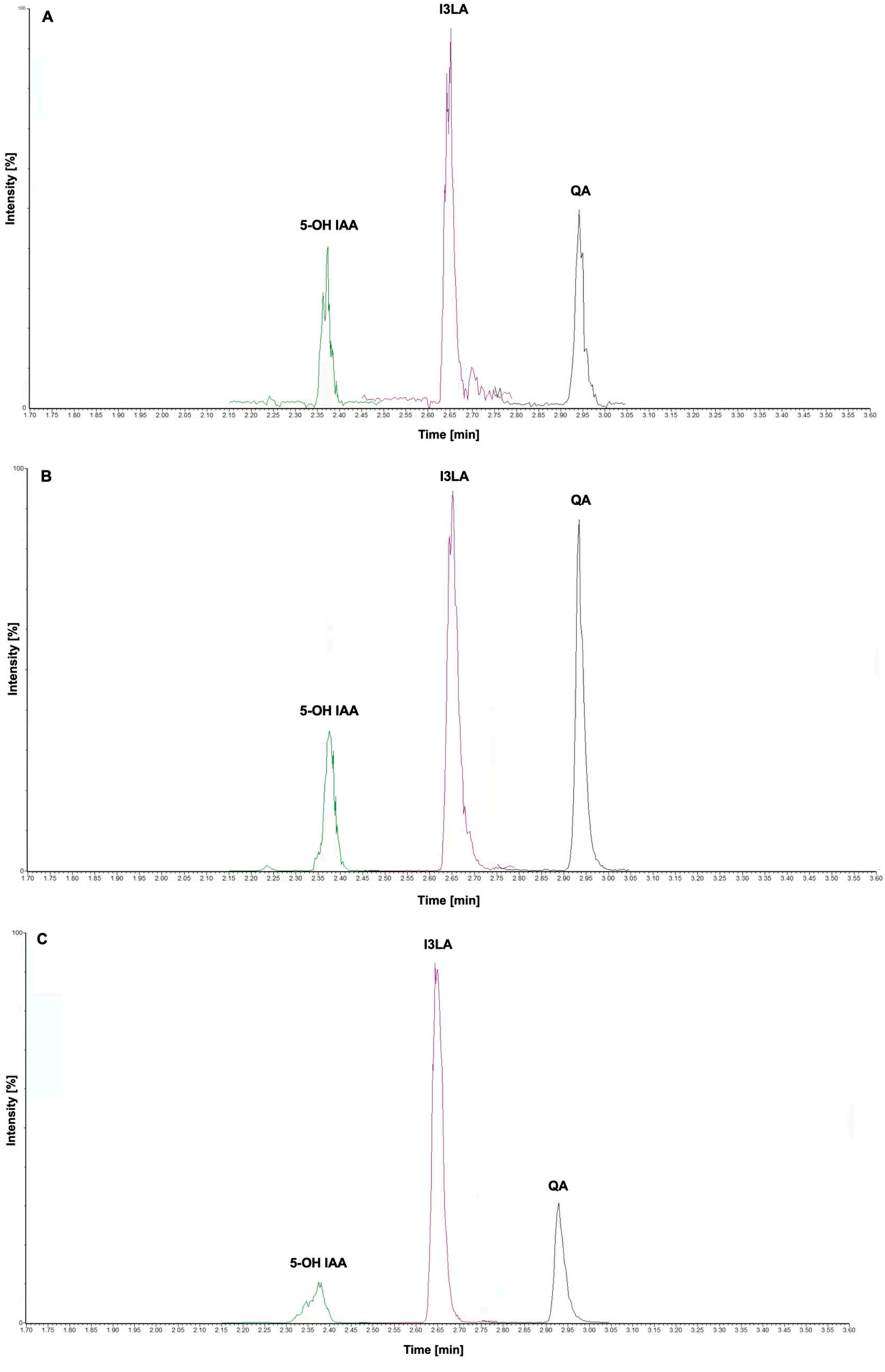

**Figure S3** Statistical evaluation of quantification of Trp metabolites in female (**A**) and male (**B**) plasma samples from AD patients and age matched CTRLs * $p < 0.05$; ** $p < 0.01$; *** $p < 0.01$; ns - nonsignificant.

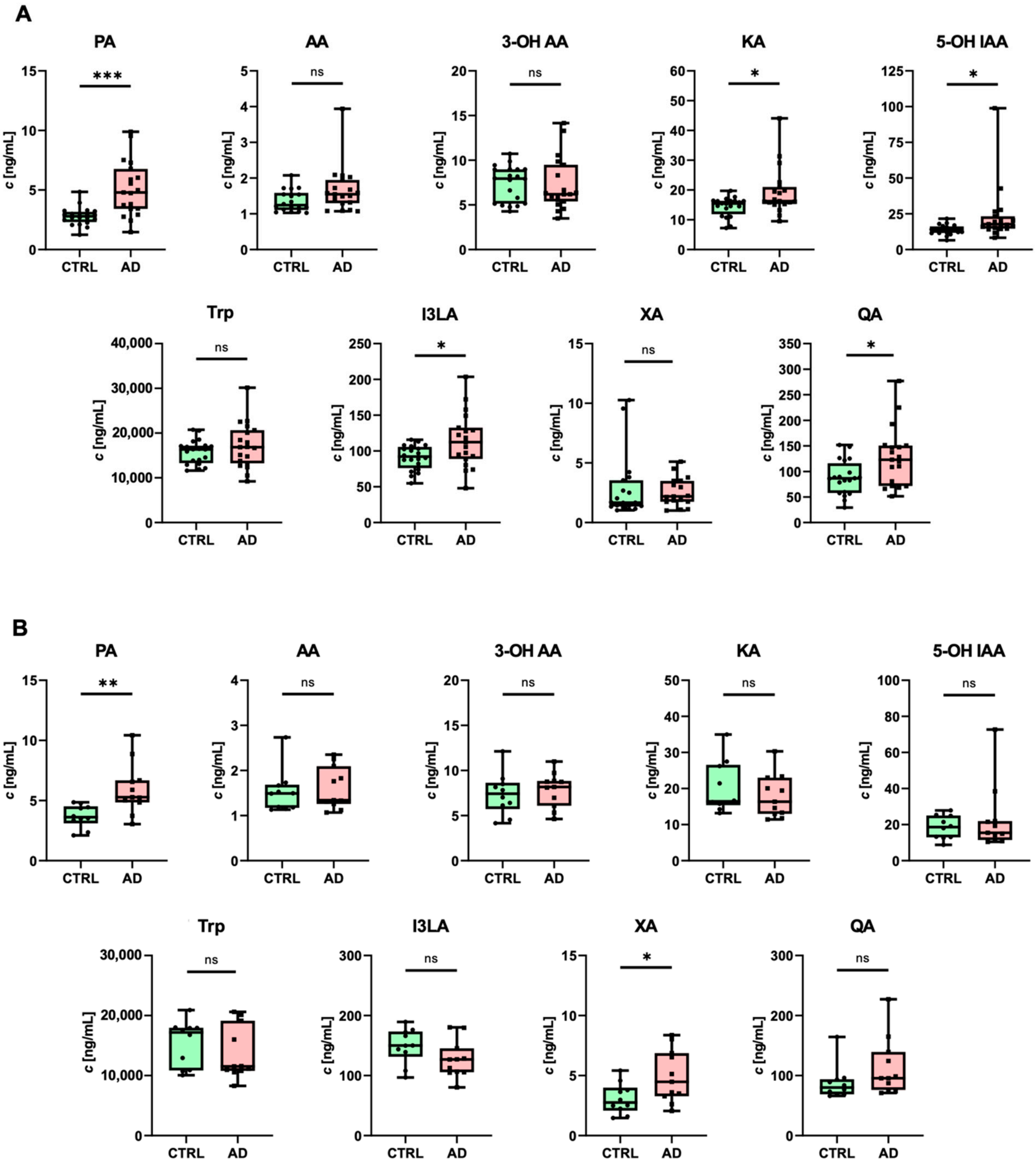

Supplement: Supplementary file 1 [file biomedicines-12-01003-s001.zip › biomedicines-2981350-supplementary.pdf]
